# A multimodal computational pipeline for 3D histology of the human brain

**DOI:** 10.1038/s41598-020-69163-z

**Published:** 2020-08-14

**Authors:** Matteo Mancini, Adrià Casamitjana, Loic Peter, Eleanor Robinson, Shauna Crampsie, David L. Thomas, Janice L. Holton, Zane Jaunmuktane, Juan Eugenio Iglesias

**Affiliations:** 1 Centre for Medical Image Computing, Department of Medical Physics and Biomedical Engineering, University College London, London, UK; 2 Department of Neuroscience, Brighton and Sussex Medical School, University of Sussex, Brighton, UK; 3 CUBRIC, Cardiff University, Cardiff, UK; 4 NeuroPoly Lab, Polytechnique Montreal, Montreal, Canada; 5 Queen Square Brain Bank for Neurological Disorders, UCL Queen Square Institute of Neurology, University College London, London, UK; 6 Neuroradiological Academic Unit, UCL Queen Square Institute of Neurology, University College London, London, UK; 7 Leonard Wolfson Experimental Neurology Centre, UCL Queen Square Institute of Neurology, University College London, London, UK; 8 Athinoula A. Martinos Center for Biomedical Imaging, Massachusetts General Hospital and Harvard Medical School, Boston, MA USA; 9 Computer Science and Artificial Intelligence Laboratory (CSAIL), Massachusetts Institute of Technology, Cambridge, MA USA

**Keywords:** Brain, Image processing

## Abstract

Ex vivo imaging enables analysis of the human brain at a level of detail that is not possible in vivo with MRI. In particular, histology can be used to study brain tissue at the microscopic level, using a wide array of different stains that highlight different microanatomical features. Complementing MRI with histology has important applications in ex vivo atlas building and in modeling the link between microstructure and macroscopic MR signal. However, histology requires sectioning tissue, hence distorting its 3D structure, particularly in larger human samples. Here, we present an open-source computational pipeline to produce 3D consistent histology reconstructions of the human brain. The pipeline relies on a volumetric MRI scan that serves as undistorted reference, and on an intermediate imaging modality (blockface photography) that bridges the gap between MRI and histology. We present results on 3D histology reconstruction of whole human hemispheres from two donors.

## Introduction

### Motivation

One of the major challenges in the quest to understand the human brain as a complex system is characterizing its multiscale organization. From a macroscopic anatomical perspective, the brain is subdivided into distinct structures and presents well-defined landmarks, for instance its gyri and sulci. At a much finer scale, neurons are interconnected to form complex circuits. These circuits give rise to features at coarser scales, e.g., the laminar organization of the cortex^1^. Although these distinct levels of organization may seem separate, they are, in fact, deeply linked and mutually influential. One clear example is given by Alzheimer’s disease, which can be described by the interactions between proteinopathy at the microscale and distributed, network-level disruptions at the macroscale^2^. Understanding these multi-scale mechanisms requires a multi-scale map of the brain. However, the current concept of brain mapping is closely linked to the specific tool used to construct cartographic representations, and thus to the spatial scale of the tool. As a result, the different organizational principles can only be observed in a scale-specific fashion with dedicated tools^3^.

Multi-scale imaging of the human brain is therefore necessarily multimodal, as different modalities are needed to study different scales. While a macroscopic anatomical picture of the brain is easily acquired non invasively or ex vivo with magnetic resonance imaging (MRI), finer characterization requires histological procedures and microscopy. Histology and MRI are highly complementary modalities: histology produces excellent contrast at the microscopic scale using dedicated stains that target different microanatomical or cytoarchitectural features, but it is a 2D modality that also inevitably introduces distortions in the tissue during blocking and sectioning. MRI does not yield microscopic resolution, but produces undistorted 3D volumes. Therefore, the combination of these two modalities offers a solution to the problem of imaging the human brain at high resolution in 3D. In fact, successful large-scale projects like BigBrain^4^ or the Allen Atlas^5^ have shown important advancements in terms of creating new whole-brain atlases with cellular-level resolution. Creating such atlases requires spatial alignment of images (“registration”) at the macroscopic (MRI) and microscopic (histology) scales. Such registration produces 3D-consistent histological volumes, and is often called “3D histology reconstruction”^6^.

Despite remarkable efforts like BigBrain, 3D histology reconstruction still presents obstacles: it requires manual intervention and tailored equipment, which leads to poor scalability and limited applicability in other experimental and clinical studies. Specifically, three main issues can be identified:

1. Cutting and sectioning of tissue for histology introduces distortions (stretching, tearing, folding, cracking, see^6^) that are specific to each section. Therefore, an external volumetric reference (typically an MRI scan) is required to produce an unbiased registration. Without such a reference, naïve pairwise registration leads to accumulation of errors (“z-shift”) and spurious straightening of curved structures (the so-called “banana effect”^7^).
2. Large differences in contrast and resolution between MRI and histology, combined with potential inhomogeneous staining and the aforementioned sectioning artifacts, make the alignment of these two modalities a difficult inter-modality registration problem.
3. The large size of the human brain, compared with most animal models, requires cutting the tissue in blocks for whole-brain analyses, with the resulting need to reassemble the blocks^6^, which is a part-to-whole registration problem^8,9^. This problem can be solved with whole brain microtomes, although such microtomes are only available in few selected sites around the world. Moreover, they exacerbate the sectioning artifacts, due to the much larger surface area of the sections.

Even when a reference MRI is available, 3D histology reconstruction is an underconstrained problem, as errors in the—typically linear—registration between the MRI and the histological stack can also be explained by nonlinearly deforming the histological sections. To overcome this problem, a common strategy is to use an intermediate modality for registration purposes. Several works^10–13^ have used photographs of the block during sectioning (so-called blockface photos). While these photos do not have nearly as much contrast or resolution as the histology, they have the advantage of being free from sectioning artifacts. Even if some nonlinear deformation remains due to tissue blocking and processing for paraffin embedding, these are negligible compared with the distortion introduced by sectioning. Therefore, blockface photographs can be corrected for illumination and perspective and then stacked into blockface volumes, which constitute a useful stepping stone between histology and MRI.

Other potential ways of obtaining 3D consistent volumes at microscopic scale include optical coherence tomography (OCT,^14^), polarized light imaging (PLI,^15^) and cleared tissue microscopy^16^. Notably, large-scale microtomes are not a feasible solution with these techniques, as they are inherently limited to small tissue blocks. In fact, despite technological advances in terms of both increasingly larger samples^17–19^ and novel microscopic acquisitions^20,21^, complete multi-scale characterization of the human brain as a whole organ still requires the use of complementary tools able to cover all the biologically relevant scales. This inherent limitation makes the development of inter-modality workflows a necessity, especially as cutting-edge research starts to target the whole human body^22^.

### Related work

There is a growing literature on the topic of combining histology with other modalities, encompassing different scopes and subdomains in medical imaging beyond neuroimaging. Here we will provide a brief survey of the approaches proposed so far for this multimodal problem, presenting first the ones focused on specific samples or small organs, and then addressing methods targeting whole organs.

Despite not being spatially comprehensive, approaches based on selected regions of interest (ROIs) have a high clinical relevance, mainly because of their potential applications in oncology. It is no surprise, then, that several studies have combined MRI with histopathology for cancer applications: examples include breast cancer^23,24^, pancreatic tumors^25^ and gliomas^26^. These examples lay the foundation for future 3D histopathology, especially given the parallel effort in 3D reconstruction for confocal microscopy^27^.

In human neuroimaging, combined MRI-histology also holds great potential because of the cross-scale nature of neurological diseases. Recent studies have proposed ROI-based approaches to better understand the microscopic substrate of pathologies such as amyotrophic lateral sclerosis^28^, epilepsy^13^ and Alzheimer’s disease^29^. The main targets mostly include subcortical structures, including thalamus^30,31^, hippocampus^32,33^, nucleus accumbens^34^, and pedunculopontine nucleus^35^, among others^10^. In addition to the practical advantage of dealing with well-defined structures, the focus on the subcortex is due mainly to its implications in neurological diseases and psychiatric disorders. Related approaches in terms of target size include methods focused on small organs. Several studies have proposed combined MRI-histology approaches for the prostate^36,37^, lymphoidstructures^38^, mammary glands^39^, and kidneys^40^. Another comparable application is the study of small animal brains, in particular rodents^41–43^ and small monkeys^44,45^.

A common element to most of the pipelines mentioned so far is the histological section stacking procedure, with registration of consecutive sections as the central step, usually using a combination of rigid and nonlinear transformations, and taking advantage of application-specific landmarks where available. An important part of these approaches is the registration between histology and MRI: choices by previous studies include use of landmarks to inform registration^28^, iterative refinement of the registration^32^, stack-based refinement^46^, and graph-theoretic stacking^33^ (for a comprehensive review, see^6^). To further facilitate the registration process, an interesting approach recently proposed by several works is based on the use of 3D printing to create personalized molds on the basis of MRI data^37,47–49^, introducing shape constraints for the subsequent cutting procedure. Specifically for brain applications^48,50^, these approaches allow to dissect the specimen in the same orientation as during the MRI scan, simplifying the subsequent matching of MRI planes and tissue slices.

In contrast to the large body of existing work in 3D reconstruction of small samples, the literature on whole-brain approaches is rather limited. Apart from the major initiatives already mentioned above^4,5^, to the best of our knowledge there are only two other studies that have targeted either the entire brain^51^ or a major portion of the cerebrum^52^.

In the studies mentioned so far, the approaches used to study microscopic features in a macroscopic context (i.e. whole-brain) can be classified on the basis of two main strategies: whole-brain sectioning, and part-to-whole registration. In studies where a whole-brain microtome was used, the section stacking procedure is similar to that sample-targeted approaches, with the main issue to tackle being z-shift and banana effects. To reduce these, it is possible to rely on blockface photographs as an additional modality^51^, multi-step registration with the MRI data^52^, or probabilistic modelling of intra- and inter-modality transformations for histology and MRI data^46^. As already mentioned, the main limitations of these approaches are the scarce availability of whole-brain microtomes, the more prominent sectioning artifacts, and the incompatibility with microscopic methods inherently based on small samples. As already mentioned, this is a significant limitation for the scalability of 3D histology, and also strongly limits attempts to leverage on new microscale technologies, as most recent advancements require small samples and therefore cutting the brain into blocks.

The other main strategy for integrating microscopic and macroscopic information targets specific brain regions using part-to-whole image registration. To tackle this problem, it is common to first initialize the ROI into rough alignment, before proceeding with finer-level registration: such initialization can be done acquiring additional MRI data after cutting the tissue sample in blocks^33^, or using photographs for each step of the cutting procedure as a collateral modality^28^. The former approach can be extended to surgical resection scenarios to relate ex vivo to in vivo data^32,53^. The main drawback for these approaches is that they are limited to specific ROIs.

### Contribution

As explained above, the number of potential approaches to probe microstructure in small samples is increasing, but directly adopting such techniques in whole human brain histology-MRI reconstruction is infeasible. Therefore, the ability to reconstruct a whole brain distribution of a given microscopic biomarker from a set of smaller samples is crucial to build multimodal, multi-scale maps of the human brain. We highlighted in the previous section how previous studies have focused either on major portions of the brain using a large-scale microtome^4,5,51,52^ or on specific ROIs using a standard microtome^10,13,28–35^. There is currently no clear approach to target a large part of the brain (e.g. a hemisphere) relying on a standard microtome. This is the gap that this study seeks to bridge: what we want to provide is an approach that does not rely on the availability of whole-brain microtomes, and that allows to study an entire human brain hemisphere, leveraging on an integrated pipeline of automated and semi-automated methods.

In this article, we present an open-source computational pipeline to reconstruct human brain volumes from histological sections with ex vivo MRI, blockface photographs and a standard microtome. To the best of our knowledge, this is the first approach able to reconstruct whole-brain 3D histology from a block-based cutting protocol. Since a highly specialized whole hemisphere microtome is not required, the proposed pipeline can be used by any research site with access to a standard microtome and an MRI scanner. The pipeline relies on a number of 2D and 3D image registration methods, some standard, and some developed specifically for this pipeline, which have already been introduced at conferences^46,54^. Here we introduce the pipeline as a whole and present results on the reconstruction of whole human hemispheres from two donors.

## Materials and methods

**Figure 1:**
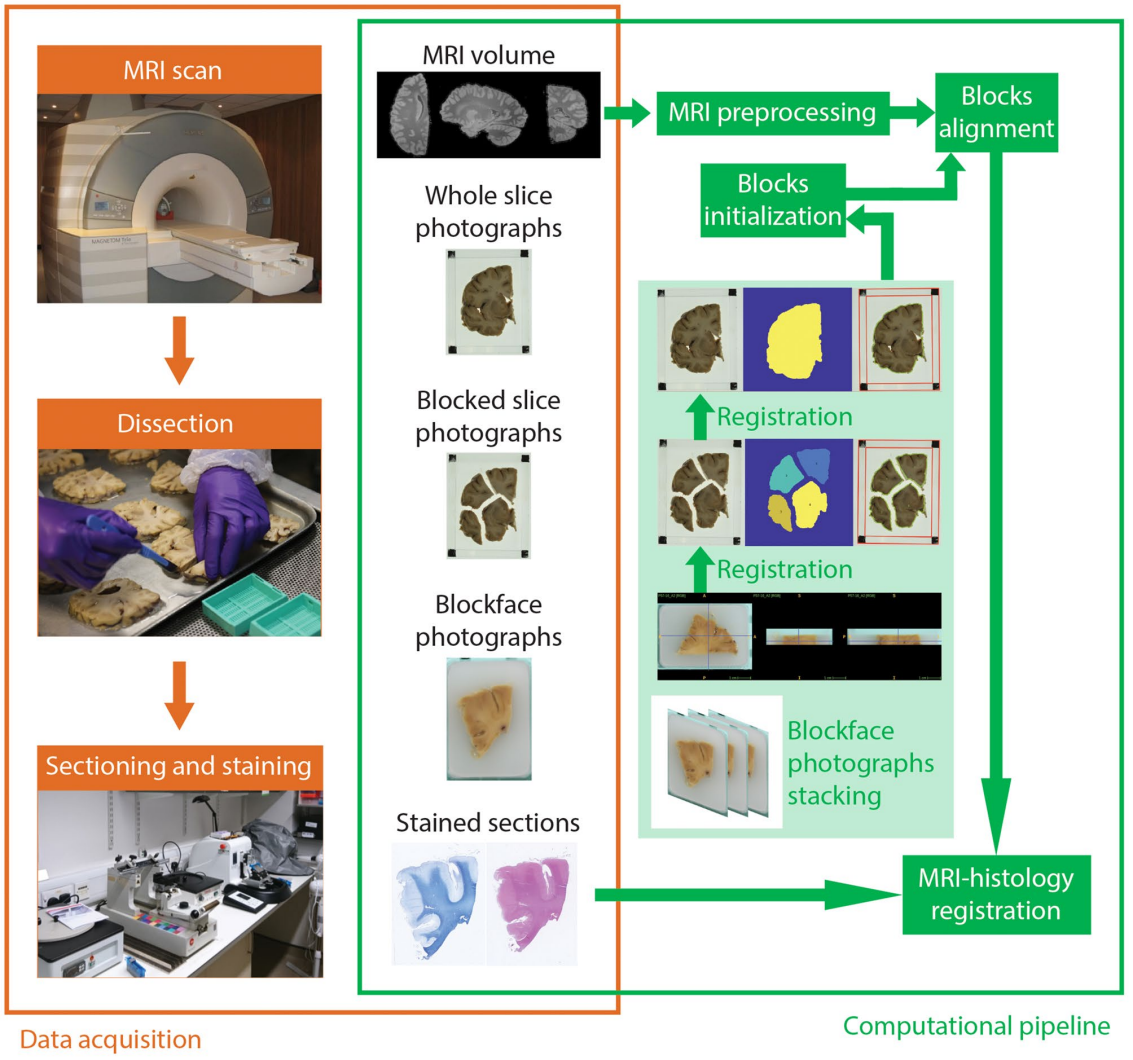
Workflow of data acquisition (orange) and computational processing (green). The ex vivo brain is scanned, dissected, sectioned and stained, providing data for the pipeline: the MRI volume, the whole and blocked slice photographs (dissection), the blockface photographs (sectioning), and the stained sections. The flowchart illustrates the main steps of the pipeline: stacking of blockface photographs; registration of blockface volumes to slice photographs; blocks initialization; block mosaic-preprocessed MRI alignment; and MRI-histology registration.

In this section, we describe the pipeline for 3D histology reconstruction, including the data acquisition protocol and computational processing, as summarized in Fig. 1. We used two human brain samples in this study. First, the left hemisphere from a human donor with no neurological disease was processed with the proposed pipeline (age at death: 84; post mortem interval: 40 h; formalin fixed whole brain weight: 1244 g). For replication purposes, the pipeline was then used to process the left hemisphere from another human donor with no neurological disease (age at death: 94; post mortem interval: 27 h; formalin fixed whole brain weight: 1200 g). All experiments were performed in accordance with relevant guidelines and regulations.

### Data acquisition

#### Specimen preparation and ex vivo MRI scanning

In this study, we use tissue donated for research to the Queen Square Brain Bank for Neurological Disorders (QSBB). The brain donation program and protocols have received ethical approval for research by the National Research Ethics Service (NRES) Committee London - Central and tissue is stored for research under a license issued by the Human Tissue Authority (No. 12198). According to the standard protocol at QSBB, fresh brains are first hemisected. The right hemisphere is frozen, while the left one is fixed in 10% neutral buffered formalin.

For ex vivo MRI scanning, it is important to immerse the brain in a fluid, in order to avoid susceptibility artifacts at tissue-air interfaces around the edges of the brain. Using the fixative as a medium for this purpose is problematic due to the high proton density of formalin, which quickly saturates the MR signal, thus greatly reducing the dynamic range of the acquired images. Instead, we use Fluorinert (perfluorocarbon), a proton-free fluid which matches the magnetic susceptibility of brain tissue but has no MR signal, so it is invisible in MR images. Immersion in Fluorinert yields excellent ex vivo contrast, and it is known not to affect subsequent histological analysis of the tissue for a wide array of stains, for up to a week of immersion^55^.

**Figure 2:**
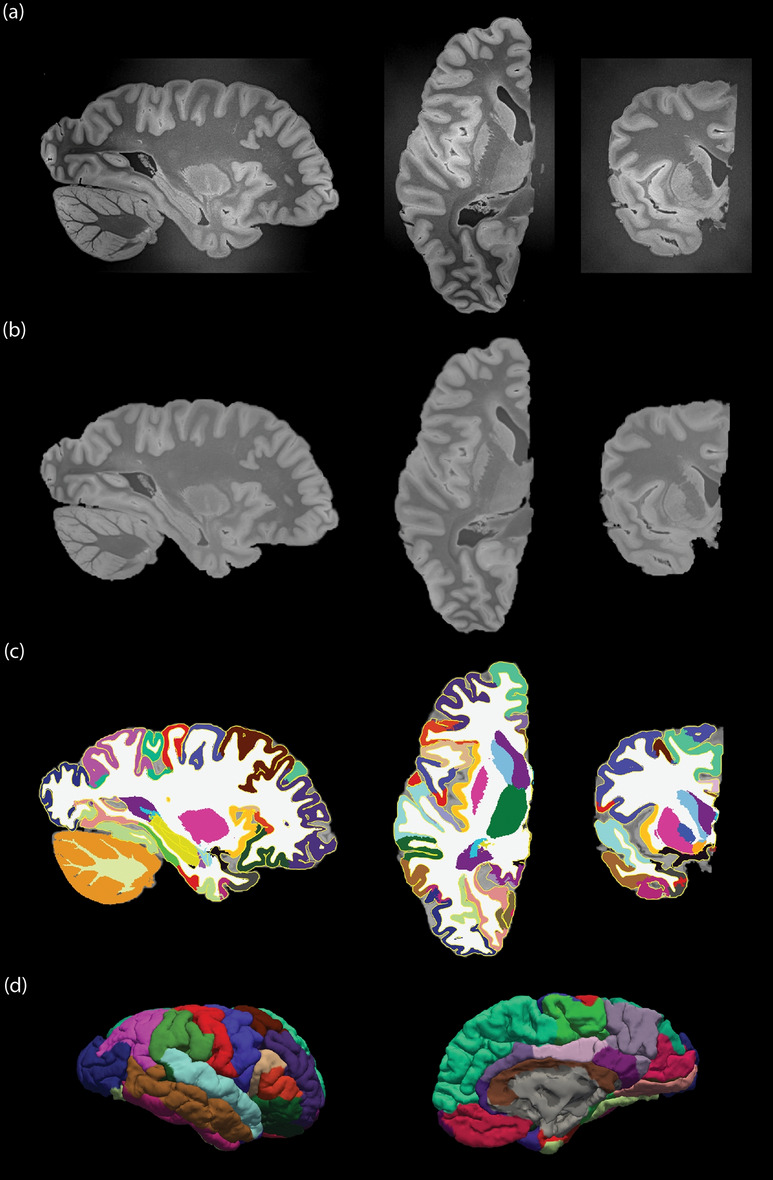
Overview of the main MRI preprocessing steps. (**a**) The raw ex vivo MRI data as acquired, showed in sagittal (left), axial (middle) and coronal (right) views; (**b**) brain extraction and bias field correction given by SAMSEG; (**c**) segmentation or subcortical structures given by SAMSEG, combined with cortical segmentation and parcellation provided by FreeSurfer, with the outline of the reconstructed surfaces in yellow; (**d**) 3D rendering of the cortical surface from lateral (left) and medical (right) views. The color coding of the segmentation and parcellation follows the FreeSurfer convention.

MRI data are acquired on a 3T Siemens MAGNETOM Prisma scanner. T1-weighted MR imaging is a common choice in vivo due to its excellent contrast between gray and white matter. However, death and fixation induce a cross linking of proteins that greatly shortens T1 relaxation times of brain tissue, reducing T1 contrast ex vivo. Instead, we use a T2-weighted sequence (optimized long echo train 3D fast spin echo,^56^) with parameters: TR = 500 ms, TEeff = 69 ms, BW = 558 Hz/Px, echo spacing = 4.96 ms, echo train length = 58, 10 averages, with 400 μm isotropic resolution, acquisition time for each average = 547 s. The choice of voxel size is motivated by the fact that 0.4 mm is enough to visualize many brain subregions, and thus suffices to serve as volumetric reference for 3D histology reconstruction^6^. Spatial variations in image signal intensity caused by the receiver array coil sensitivity profiles are accounted for using a vendor-provided online correction (Prescan Normalize). The hemisphere is scanned in a container filled with Fluorinert, with the medial surface facing up. We place a 3D printed hollow box between the specimen and the container lid in order to ensure full immersion in the fluid. Sample slices of the acquisition are shown in Fig. 2a.

#### Specimen dissection and slice photography

**Figure 3:**
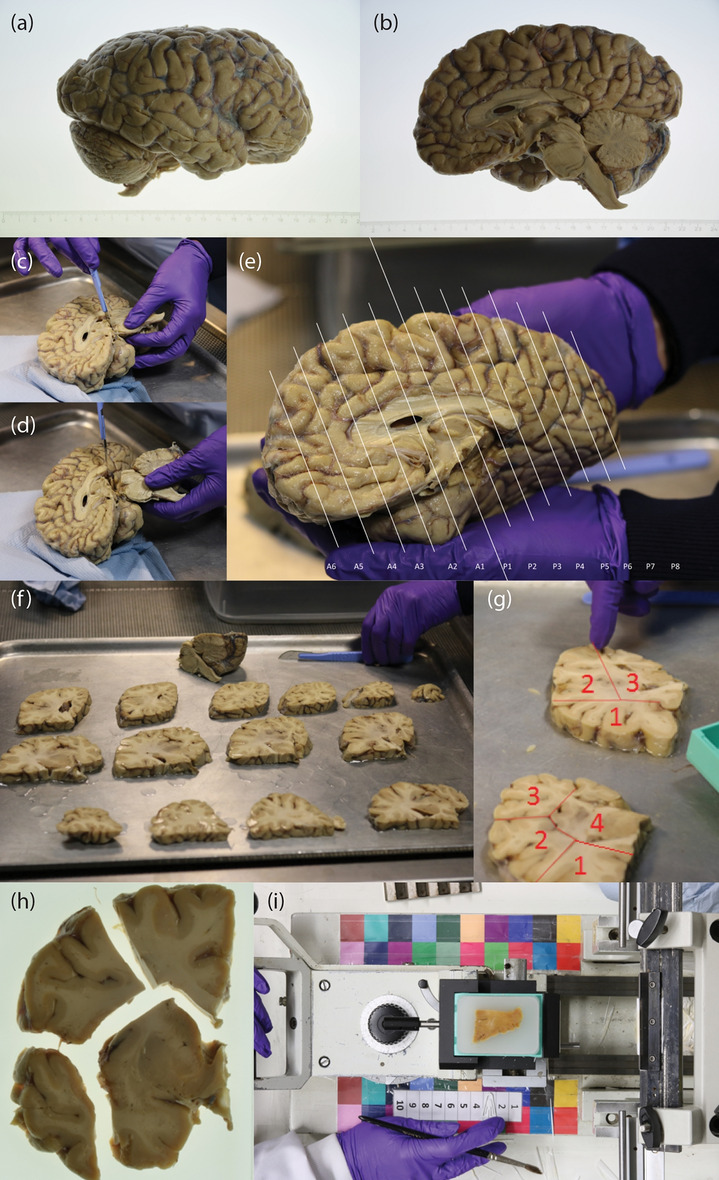
Overview of the dissection procedure. (**a**, **b**) Brain hemisphere from lateral (**a**) and medial (**b**) views; (**c**) incision; (**d**) subsequent brainstem transection below the mamillary body; (**e**) overview of how the anterior and posterior slices are cut: the forebrain is cut coronally; (**f**) forebrain slices and remaining brainstem and cerebellum portions: the brainstem is cut transversely while the cerebellum is cut sagittally; (**g**) example of block cut planning; (**h**) example of blocked slice photograph; (**i**) example of blockface photograph, including the microtome setup.

After MRI scanning, the hemisphere is dissected following the procedure illustrated in Fig. 3. First, the brainstem is detached with a transection below the mammillary body, using as a plane of section the one perpendicular to the brainstem axis (Fig. 3c,d), and the cerebellar hemisphere is separated from the brainstem by carefully cutting through the superior, middle and inferior cerebellar peduncles. The three structures are then dissected independently. The forebrain is first cut into 10 mm-thick coronal slices using a metal frame as a guide (frame thickness: 10 mm), starting from the mammillary body and proceeding in both anterior and posterior directions (Fig. 3e,f). In a similar way, the cerebellum and the brainstem are sliced in sagittal and axial orientation, respectively, with the same thickness. All the slices are then photographed on both sides (posterior and anterior for the forebrain, rostral and caudal for the brainstem, medial and lateral for the cerebellum) inside a rectangular frame of known dimensions (internal boundary: 120 mm $\times$ 90 mm), and thickness equal to the slice thickness (10 mm). The frame enables perspective correction and pixel size calibration in subsequent steps of the pipeline. We will refer to these images as “whole slice photographs”, which will be useful to initialize the 3D histology reconstruction.

The forebrain sections are further cut into blocks that fit into 74 $\times$ 52 mm cassettes, seeking to minimize the number of blocks while trying to preserve the integrity of subcortical structures (Fig. 3g). In our datasets, cutting the brainstem and the cerebellum in blocks was never necessary, since they always fit directly into the cassettes. Photographs of the blocked slices, where the blocks were slightly pulled apart to clearly expose their boundaries, were taken using the same frame as for the whole slices, both from the anterior and posterior side. We will refer to these images as “blocked slice photographs”.

All slice photographs (whole and blocked) are taken with a Nikon D5100 camera mounted over the samples. Consistent image contrast across samples is ensured by manually setting: ISO = 100; one-shot auto focus using a single point in the center of the image (which is aligned with the center of the slice); and f/20 aperture for large depth of field. The shutter speed was computed automatically by the camera to compensate for variations in lighting level. Examples of slice photographs are shown in Fig. 5a,b.

#### Tissue processing, sectioning and blockface photography

All blocks are processed for paraffin wax embedding, and subsequently sectioned with a sledge microtome at 25 μm thickness.

Minor artifacts such as tissue folding and tearing occur during this process, but they were never severe enough to impede the subsequent steps in the pipeline.

Before cutting each section, a photograph is taken with a camera mounted above the microtome, set in a fixed position that is approximately perpendicular to the slicing plane (Fig. 3i). We will refer to these images as “blockface photographs”. Since these photographs will need to be perspective corrected, pixel size calibrated, and co-registered (since keeping the camera absolutely still is not possible), we printed and glued two checkerboard patterns with maximally distinct colors^57^ to the microtome, which facilitates subsequent registration. The photographs are taken at 24MPx resolution with a Canon EOS 750D camera. As for the slice photographs, we use manual settings to ensure consistency of image appearance across sections: ISO = 200; white balance = fluorescent light source; one-shot auto focus using a single point in the center of the image (which is aligned with the center of the tissue block); and crucially, a narrow aperture (f/13) for large depth of field, thus ensuring sharpness of objects not exactly in focus. An example of blockface photograph is shown in Fig. 5c.

#### Staining and digitization

The sampling strategy for mounting and staining sections for each tissue block was tailored to the presence of structures of interest for post-mortem neuropathology. In line with previous studies^10,30–35^, a more dense sampling scheme was used for blocks that included subcortical structures in the forebrain, medial structures of the cerebellum and brainstem structures. Specifically, for such blocks, we mount on glass slides and stain two consecutive sections every 10 sections (i.e., every 250 μm) with two routine histological stains: hematoxylin and eosin (H&E) and Luxol Fast Blue (LFB). We use an H&E protocol with short haematoxylin staining time, which is optimized for microscopic examination, but yields less macroscopic contrast between gray and white matter—this does not constitute a problem since such contrast is guaranteed by LFB staining. For the other blocks, the frequency is one every 20 sections instead (i.e., every 500 μm). The sections are mounted on 75 $\times$ 50 mm glass slides. We also mount 2 additional slides every 10 (blocks of interest) or 200 sections (other blocks), kept unstained for potential future use. With this sampling strategy, the resulting transverse (i.e. across sections) resolution when reconstructing 3D blocks is either 250 μm or 500 μm: these values are assumed to be adequate for the size of most cortical and subcortical structures.

Stained sections are digitized with a flatbed scanner (Epson Perfection V850) using its transparency mode at 6,400 DPI (i.e., 3.97 μm resolution). This resolution is sufficient for 3D histology reconstruction purposes. Selected sections are also digitized at microscopic resolution (40 $\times$) using an Olympus VS120 microscope/slide scanner, and linearly aligned to the corresponding images acquired with the flatbed scanner using NiftyReg^58^.

### Computational pipeline

**Figure 4:**
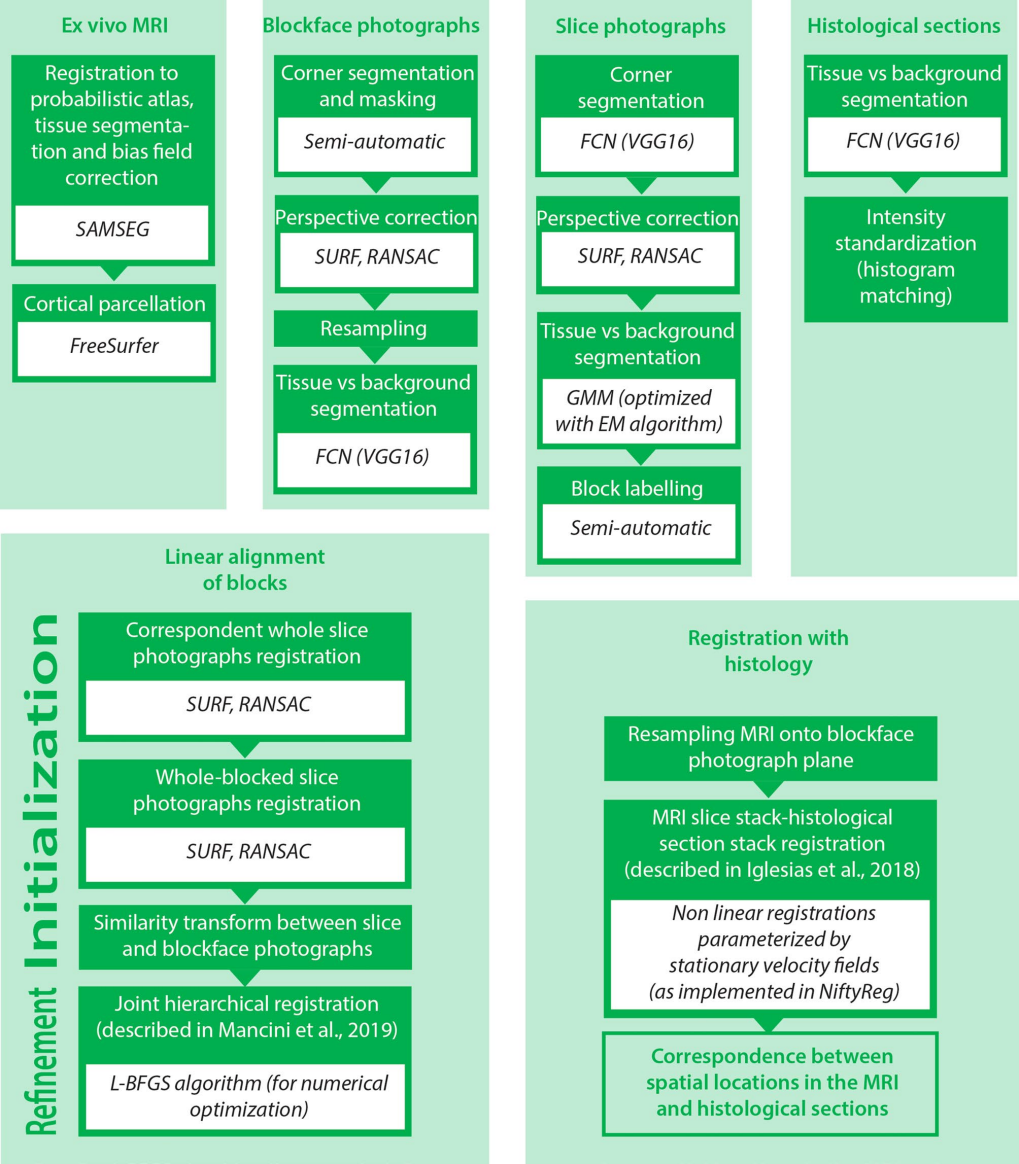
A flowchart of the computational pipeline. This flowchart represents the main processing steps, subdivided by the related subsections in the main text. Where relevant, the flowchart specifies what algorithm or software was used, or if any manual intervention was needed.

After completing the acquisition of MRI and histological data, the following pipeline is used to compute the 3D reconstruction. The following sections are briefly summarized in Fig. 4.

#### Ex vivo MRI

The T2-weighted MRI scan is preprocessed using a Bayesian segmentation algorithm (SAMSEG^59^) that simultaneously registers, segments and bias field corrects the scan (Fig. 2b). To reflect the presence of just the left hemisphere in the images, we modified the SAMSEG probabilistic atlas by manually setting to 1 the probability of background (and to 0 for all other classes) for all voxels in the right half of the atlas. Our modified SAMSEG produces a bias field corrected scan, as well as segmentations for 22 brain structures: cerebral white matter, cerebellum white matter, brainstem, ventral diencephalon, optic chiasm, cerebral cortex, cerebellum cortex, caudate, hippocampus, amygdala, accumbens area, lateral ventricle, inferior lateral ventricle, third ventricle, fourth ventricle, fifth ventricle, cerebrospinal fluid, vessel, choroid plexus, thalamus, putamen, and pallidum.

After SAMSEG, we used the FreeSurfer^60^ to extract and parcellate the cortical ribbon. Specifically, we used the ex vivo processing stream described in freesurfer.net/fswiki/ExVivo (Fig. 2c,d): (i) computation of a white matter mask (rather than using the expectation maximization algorithm described in the FreeSurfer wiki, we directly extract the mask from the SAMSEG segmentation); (ii) extraction of a triangular mesh from the cerebral white matter segmentation with marching cubes^61^; (iii) inflation of the mesh and mapping to spherical coordinates^62^; (iv) topology correction^63^; (v) reconstruction of white matter and pial surfaces^64^; and (vi) cortical parcellation^65^. While parcellation is not strictly required by our reconstruction methods, it can be used to subdivide the cerebral cortex in the histology, which would be extraordinarily difficult for a human labeler.

**Figure 5:**
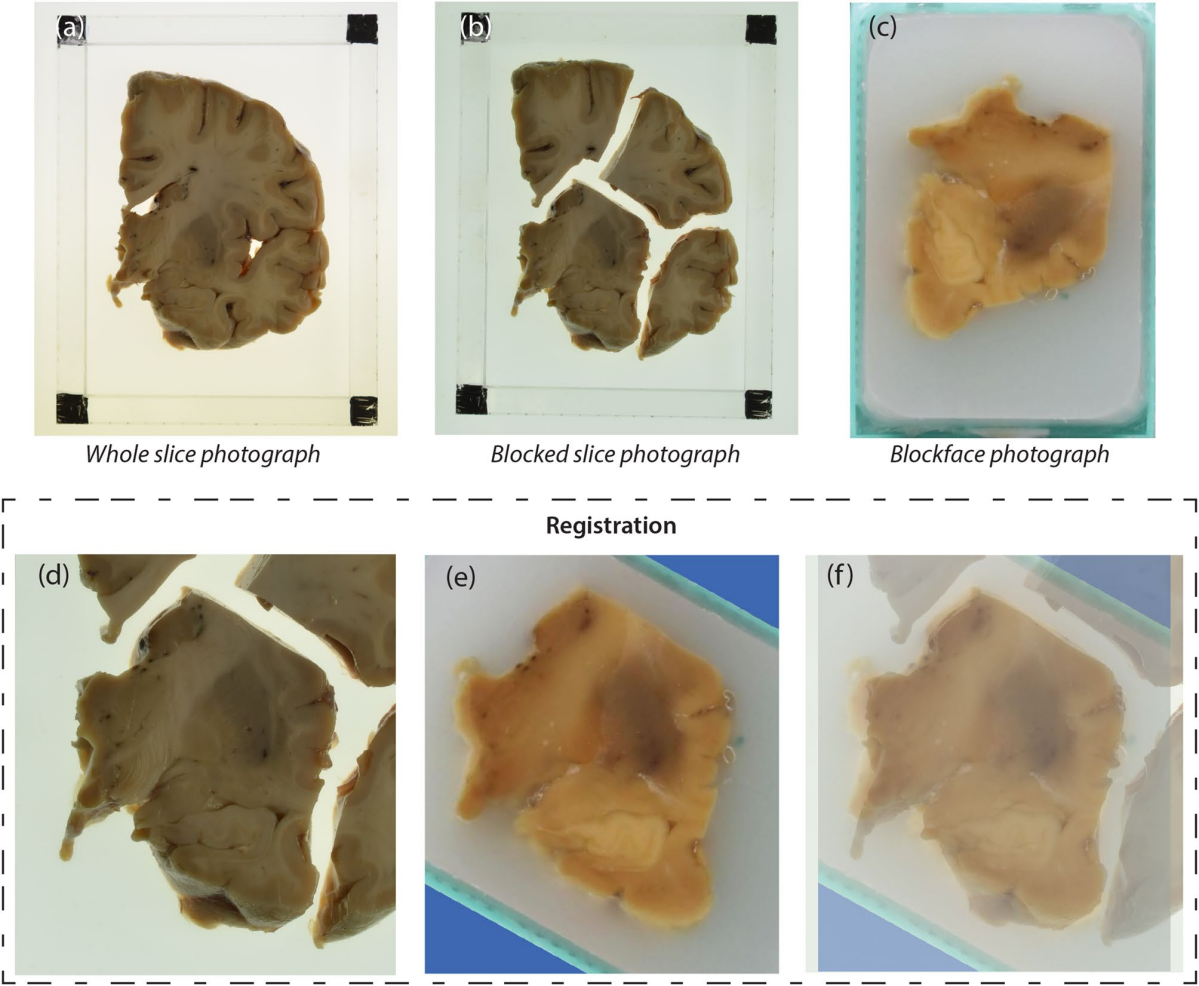
An example of blocked slice photograph to blockface photograph registration. The three images on the top show the three types of photograph we are dealing with: (**a**) a sample whole slice photograph; (**b**) the related blocked slice photograph; (**c**) corresponding blockface photograph of the bottom left block. The three images on the bottom show the outcome of the registration: (**d**) block of interest in the blocked slice photograph; (**e**) aligned blockface photograph; (**f**) blocked slice photograph with blockface photograph overlaid in transparency.

#### Blockface photographs

In an ideal scenario, the blockface photographs would be perfectly aligned without any need for processing. However, the position of the arm holding the camera can suffer from small perturbations due to vibrations in the furniture and walls, operation of the microtome, and other external factors. Therefore, it is necessary to align the photographs before further processing. For this purpose, we first create a global reference image (“microtome reference”), which is a photograph of the microtome with an empty cassette. On this image, we manually mark the four corners of the cassette, and delineate two masks: one over the checkerboard patterns, and another over a band around the edges of the cassette.

We use the microtome reference to perspective correct and calibrate the pixel size of all other blockface photographs, by propagating the location of the four corners of the cassette. For this purpose, we first select the photograph half way through the block, which we will refer to as “block reference”. The microtome reference is registered to the block reference to propagate the location of the cassette corners as follows: (i) we compute salient points and SURF features^66^ on both images, and discard those outside the checkerboard in the microtome reference; (ii) we match the salient points; (iii) we use random sample consensus (RANSAC,^67^) to fit an homography (perspective) transform; and (iv) we refine the registration to accurately align the cassettes, by repeating the procedure in steps (i-iii), but with two differences: we use the mask for the cassette edges instead of the checkerboard mask, and we add an Euclidean distance term to the matching, since the cassettes are already in coarse alignment. We use RANSAC to make the registration robust against different positions of the microtome handle and appearance of the tissue block. The final transform is used to propagate to the block reference the location of the manually labeled cassette corners, as well as the mask for the checkerboard patterns.

Once we have estimated the checkerboard mask and cassette corners for the blockface reference, we register all photographs in the block to the reference using steps (i-iii) of the procedure described above. Finally, we compute an homography transform between the four cassette corners and coordinates (1, 1), (1, 740), (520, 1), (520, 740), and use it to resample the blockface photographs into 520 $\times$ 740 pixel images with known pixel size equal to 100 μm, where the corners of the image coincide with the corners of the cassette. These images can be safely stacked into a single volume, with *z* resolution equal to 25 μm. We note that the in-plane pixel size is slightly overestimated due to the fact that the actual blockface is slightly closer to the camera than the cassette. Moreover, the *z* resolution is also corrupted by inaccuracies in the section thickness provided by the microtome. Nevertheless, these voxel size errors do not represent a problem in practice because tissue shrinks during processing, and both the pixel size and section thickness need to be corrected in subsequent steps of the computational pipeline anyway.

The blockface photograph module is completed by a supervised segmentation algorithm, which discriminates tissue versus background wax.

Here we use a fully convolutional network (FCN) for semantic segmentation^68^. Compared with classification networks that assign a label to a whole image, FCNs only use convolutions and can automatically segment inputs of arbitrary size, producing outputs of the same size. Moreover, they can be built directly on top of existing image classification networks, thus capitalizing on existing models trained on million of examples. Specifically, we use the VGG16 architecture^69^, pretrained on the large public dataset ImageNet^70^. The FCN is trained with manual segmentations made on 50 randomly selected (perspective corrected, pixel size calibrated) images from different blocks. The manual segmentations aggressively discard tissue below the block surface (which appears blurry due to the translucent paraffin), such that the FCN attempts to mimic this behavior and thus avoids overestimating tissue surface in convex blocks. While the FCN operates in 2D, stacking the automated segmentations yields a 3D mask that is spatially smooth, due to the 3D smoothness of the underlying images.

#### Slice photographs

The slice photographs are crucial to initialize the registration of the tissue blocks. Processing of both whole and blocked slice photographs begins by segmenting the corners of the frame, which are painted in black, using a FCN similar to the one used for blockface photographs. This time we used 20 manually labeled images for training, which is enough, given the simplicity of the problem. The center of gravity of the four largest clusters are identified as the corners. Then, four sets of parallel lines are fit to the gradient magnitude images, to identify the internal and external boundaries of the frame. The internal corners are computed as the intersections of the internal boundaries, and used to fit an homography to correct for perspective and calibrate the pixel size to 100 μm (i.e., 1,200 $\times$ 900 pixels), in a similar way as for the blockface photographs.

Next, the blocked slice photographs (perspective and pixel size calibrated) are segmented into foreground and background using a simple Gaussian mixture model (GMM) with two components, optimized with the Expectation Maximization (EM) algorithm^71^. The resulting mask is overlaid onto the corresponding image and displayed on a simple graphical user interface (GUI), where a user assigns block numbers to the different connected components of the binary mask, producing a multilabel segmentation of the different blocks.

#### Digitized histological sections

Processing of digitized histological sections has two components: segmentation and intensity standardization. For segmentation, we used two FCNs, one for LFB and one for H&E, trained on 50 randomly selected sections each. Simple intensity standardization was carried out using only the pixels inside the masks, by matching their histogram to the average histogram of the foreground pixels of the training dataset.

#### Linear alignment of blocks

Direct nonlinear registration of 2D histological sections to a volumetric reference (an MRI scan in our case) is a highly underconstrained problem^6^, whereas linear registration is well constrained but also insufficient to model the heavy spatial distortions introduced by tissue sectioning and mounting. This is the motivation for using blockface photography as intermediate modality. Even though some nonlinear deformation still remains due to sample slicing and blocking, as well as tissue processing for paraffin embedding, imaging the blockface before sectioning removes the largest contribution to nonlinear distortion. Therefore, the simplified assumption of the spatial mapping between blockface photography and MRI being linear is approximately valid.

In our pipeline, we first use the slice and blockface photographs to initialize the registration between blockface volumes and the MRI. Then, we use a dedicated joint registration algorithm to optimize the alignment. To initialize the registration, we start by calculating three different sets of 2D linear transforms. First, we use SURF and RANSAC (see section “Blockface photographs”) to rigidly register whole slice photographs of the lateral/anterior/inferior face of each block to the medial/posterior/superior face of the neighboring lateral/anterior/inferior block. These images are nearly identical, so registration is easy and accurate. Second, we use SURF/RANSAC to rigidly align each block in the blocked slice photographs (using the available multi-label masks) to the corresponding whole slice photograph. Despite being a whole-to-part registration problem, SURF/RANSAC produces accurate solutions, since the photographs are of the same objects and acquired in the same illumination conditions. And third, we estimate a similarity transform (i.e., translation, rotation and scaling) between each block in the blocked slice photographs and the approximately corresponding image in the blockface photograph volume, using mutual information as cost function^72–74^. The target blockface photograph is the first one where the whole block is completely visible, which we estimate by finding the first section in which the ratio between surface area and its maximum across the block is at least 2/3. The surface areas are estimated with the masks produced by the FCN. An example of the alignment between a blocked slice photograph and the related blockface one is showed in Fig. 5d–f.

Given these sets of 2D transforms, initializing the blocks in the cerebellum, forebrain and brainstem is straightforward. For the forebrain, a reference slice is first chosen (the one corresponding to the mammillary bodies, in our case). Then, for each block, one simply concatenates its corresponding blocked-slice-to-whole-slice transform, along with all the whole-slice-to-whole-slice transforms between the slice at hand and the reference. Finally, a shift in the anterior–posterior direction is computed by each block, which is simply equal to the slice thickness (10 mm) multiplied by the (signed) number of slices between the slice at hand and the reference. The cerebellum and brainstem are processed the same way, with two differences: the blocked-slice-to-whole-slice transform is not needed (since slices are not blocked), and the shifts occur in the medial–lateral (cerebellum) and inferior–superior directions (brainstem). Finally, the three sets of blocks (forebrain, cerebellum and brainstem) are manually aligned to the preprocessed reference MRI using a rigid transform by means of tools available in Freeview (FreeSurfer’s volumetric image viewer).

Once the transforms for each block have been initialized, they are optimized using a joint hierarchical registration algorithm. The details of the methods can be found in^54^, but we summarize them here for completeness. Each blockface volume has an associated spatial transform that has a set of 8 parameters, corresponding to 3D translation (3 parameters), 3D rotation (3), in-plane scaling (1), and scaling along the thickness direction (1). The cost function of the registration combines a data term and two regularizers. The former is simply the correlation of edge maps. The first regularizer is a customized penalty term that encourages the sum of the soft deformed masks to be equal to the binary mask of the MRI. This regularizer penalizes gaps between blocks (the sum is zero, whereas the target is 1) as well as overlapping blocks (sum is 2, target is 1), and also encourages the surface of the whole hemisphere to be the same for the MRI and the mosaic of blockface volumes. The second regularizer penalizes deviations of the global scaling of each block (i.e., the cubic root of the determinant of its linear transformation matrix) from an empirical value, which represents the expected tissue shrinkage, and which we derive from the blocked-slice-to-blockface registrations.

The optimization procedure is hierarchical, in order to exploit prior knowledge on the cutting procedure. There are five levels of hierarchy. At the first level, blocks are grouped into three sets (forebrain, cerebellum, brainstem), each of which undergoes an independent rigid registration. At the second level, the forebrain blocks are grouped into corresponding slices and can only rotate or translate simultaneously in the slice plane. At the third level, individual translation and rotation are allowed, with the addition of a scaling factor that is common to all blocks. At the fourth level, each block is allowed its own scaling factor. At the fifth and final level, each block has its own transform. We use the L-BFGS^75^ algorithm for numerical optimization of the cost function.

#### Registration with histology

Once the blockface volumes have been linearly registered to the reference MRI volume, we can use the resulting transforms to resample the MRI to the planes corresponding to the different blockface photographs—and therefore the histological sections, since we know what photograph corresponds to each section.

Furthermore, the 2D resampled MRI slices can be masked with the automated segmentations of the blockface photographs provided by the FCN. This process yields a stack of resampled, segmented MR images that have direct correspondence to the images in the histology stack.

To 3D reconstruct the histology for each of the two stains (LFB and H&E), we used a method that we presented in^46^, and which we summarize here for completeness. The goal is to register a stack of histological sections to a corresponding stack of resampled 2D MRI slices. First, the histology stack is put into coarse alignment by linearly registering each section to the corresponding MR image. For this purpose, we used the linear registration module in NiftyReg^58^, which relies on a block matching approach and mutual information. Then, we compute a set of nonlinear registrations parameterized by stationary velocity fields (SVF,^76^), as implemented in NiftyReg. Using SVFs has three advantages. First, the corresponding deformations are guaranteed to be diffeomorphic and thus invertible; second, inversion is achieved simply by changing the sign of the SVF; and third, composition of transforms can be approximated by the sum of the corresponding SVFs. The set of registrations includes: (i) inter-modality registrations between each histological section and the corresponding MRI slice, computed with mutual information; (ii) intra-modality registrations, between each histological section and its two nearest neighbors in the stack, computed with local normalized cross-correlation; and (iii) intra-modality registrations, between each MRI slice and its two nearest neighbors in the stack, also computed with local normalized cross-correlation.

While one could use the inter-modality registrations—i.e., subset (i)—directly to obtain a 3D reconstruction, this approach is known to produce volumes that are jagged, due to inconsistencies in the registrations of neighboring image pairs. At the opposite end of the spectrum, an alternative approach is to use only the intra-modality registrations for histology, i.e., subset (ii), but this method leads to accumulation of errors along the stack (“z-shift”) and straightening of curved structures (“banana effect”, see^6^). Instead, our method^46^ achieves the best of both approaches by combining all three subsets. The method relies on a spanning tree of unknown, “true” deformations connecting all the images in the two stacks. Then, all the registrations in the three subsets can be seen as noisy measurements of compositions of transforms in the set of true deformations. Within this model, Bayesian inference is used to compute the most likely set of underlying true deformations that gave rise to the observed data. This approach produces 3D reconstructions that are both smooth and robust against z-shift and banana effect. The intuition behind it is that subset (i) aligns the two stacks; subset (ii) ensures the smoothness, and subset (iii) enables us to undo the banana effect incurred by subset (ii).

After running all the modules of the computational pipeline, correspondence between spatial locations in the MRI and digitized histological sections can be obtained by concatenating 3D (blockface volume to MRI) and 2D spatial transforms (histology to resampled MRI).

## Results

In this section we present intermediate and final results, including 3D tissue blocks, alignment with MRI and 3D histology reconstruction.

### Building blockface volumes

**Figure 6:**
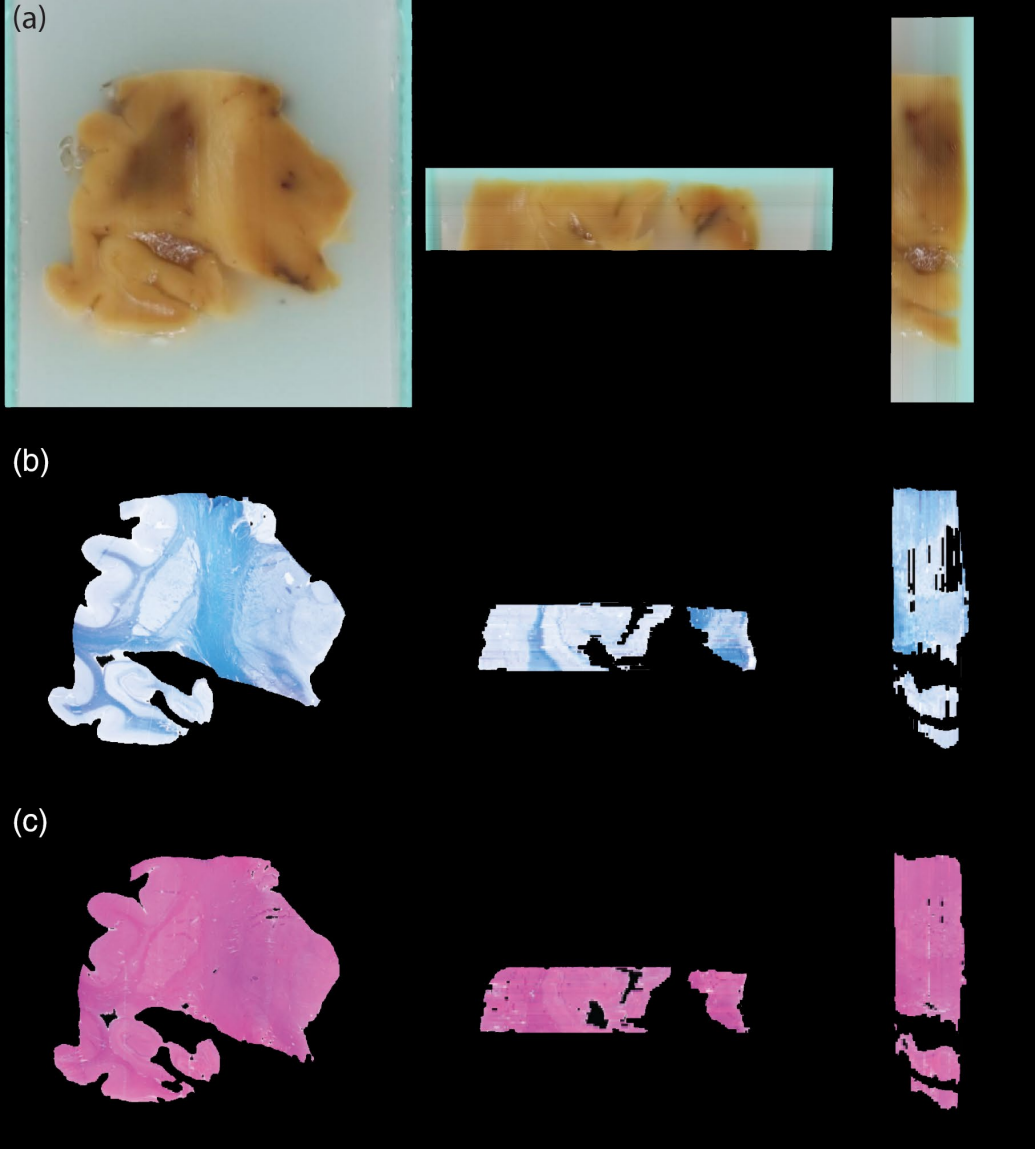
Blockface and histological volumes. Example of reconstructed blockface volumes (**a**) from three different views (coronal on the left, axial in the centre, and sagittal on the right). The same views are also showed for the correspondent histological volumes, stained with LFB (**b**) and H&E (**c**).

Figure 6 displays results from the first fundamental step of our 3D histology pipeline: assembling blockface photographs into 3D volumes. The reconstructed views of a sample cerebral block (axial and sagittal, Fig. 6a) present a smooth and consistent profile, demonstrating how effective the co-registration strategy we adopted is.

Figure 6b,c provides a first glimpse of how the blockface volumes are used as a stepping stone in the overall process of fusing MRI and histology. The figure shows the histological sections (LFB and H&E) corresponding to the blockface photograph in Fig. 6a. For easier visualization, we show the sections after nonrigid registration with the MRI, which indirectly aligns them with the blockface photograph as well. Therefore, the volumes are consistent with each other—but they also shows some differences because of inevitable artifacts that occur during sectioning.

### Effective alignment of MRI reference and tissue blocks

**Figure 7:**
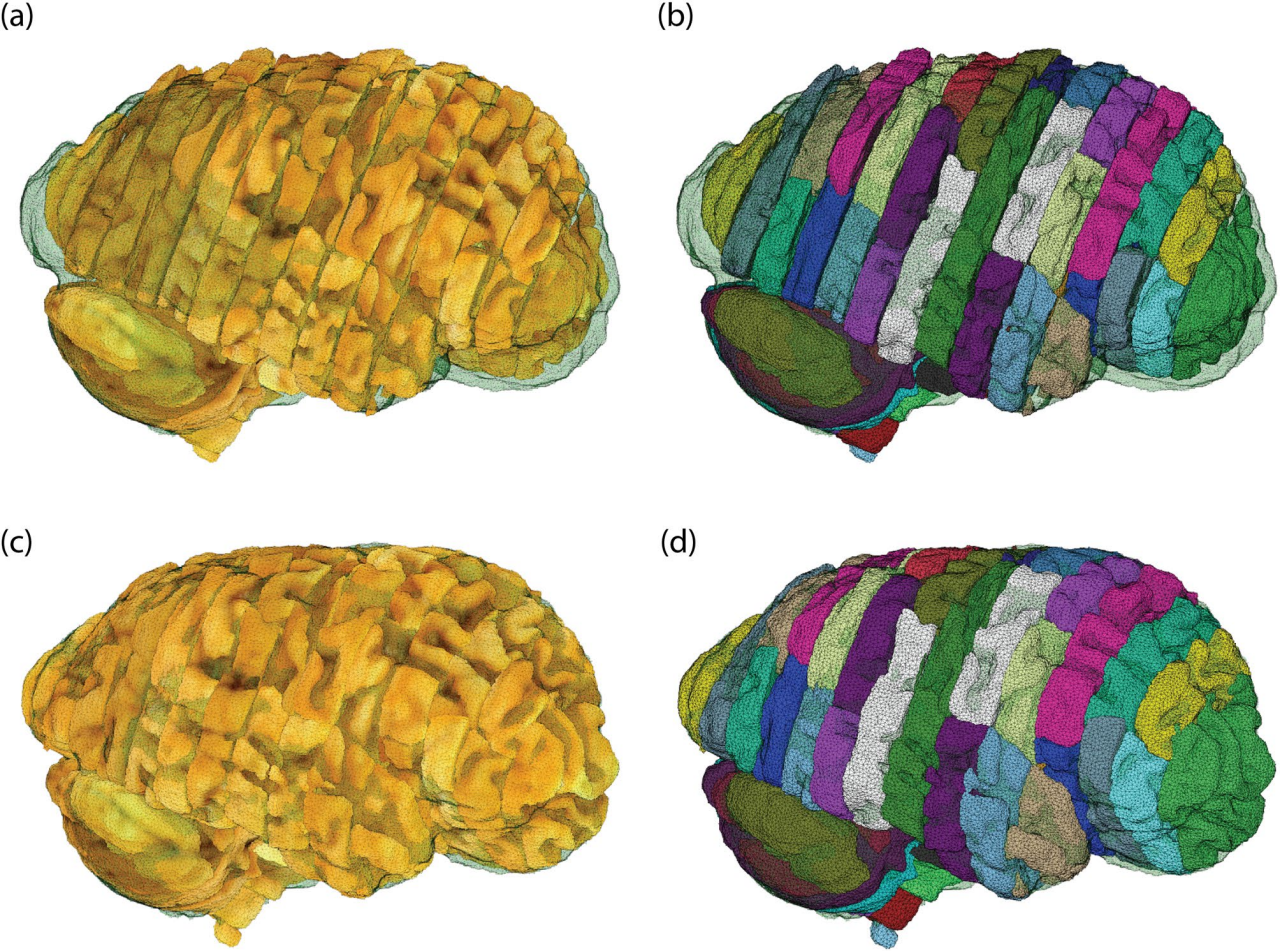
Initialization and refined blockface mosaics. A surface rendering of the whole brain mask derived from the MRI is overlaid in green. (**a**) Blockface volume for the whole hemisphere resulting from the initialization with the slice photographs; the color for rendering is taken from the blockface photographs. (**b**) Same volume as (**a**), where each block is rendered in a different color. The color coding is random and simply emphasizes the block cutting profiles. (**c**,**d**) Same volumes as (**a**,**b**) after joint registration.

Once all the blockface volumes have been assembled and initialized, a 3D mosaic representing the brain is obtained (Fig. 7a,b). Notably, the overall shape and the coarse subdivisions (forebrain, cerebellum, brainstem) are a good first approximation, although misalignment in the gyrification patterns and in the antero-posterior organization are clearly noticeable. When compared with the actual MRI reference (whose whole brain mask is surface rendered in transparent green), there is a clear mismatch in their shapes.

Our joint registration procedure yields the refined mosaic shown in Fig. 7c,d: not only the blocks match the MRI reference well, but also yield a more consistent and smoother gyrification pattern. Similar results were obtained for the second hemisphere (Supplementary figure 1). Supplementary video 1 thoroughly illustrates the joint registration procedure, and gives a more detailed overview of both the initialized and the refined mosaics—highlighting how the consistency of brain structures improves as a result of the spatial alignment.

### Nonlinear alignment of MRI and histology

Concatenating the linear alignment of blocks (“Linear alignment of blocks”) with the nonlinear registrations of the histological sections yields a complete nonlinear 3D histology reconstruction. While the two registration algorithms have been quantitatively validated in the corresponding publications^46,54^, here we seek to evaluate the accuracy of their concatenation. For this purpose, we manually annotated 50 corresponding pairs of landmarks on both modalities for each hemisphere, and compared the ground truth shift with the estimate given by the registration. We note that we used continuous coordinates, i.e., the landmarks are not constrained to lie in the center of voxels. This experimental setup enables us to measure registration error directly, although it suffers from two sources of bias. The first is due to the fact that human observers generally choose salient points (i.e. prominent anatomical features) as landmarks. Because salient points are normally easier to register, they often underestimate registration error. The second bias is due to the fact that annotating landmarks in 3D is very difficult and subject to relatively large inter- and intra-observer variability (a few mm^77^), hence overestimating registration error—and partly compensating the positive bias due to landmark placement.

**Figure 8:**
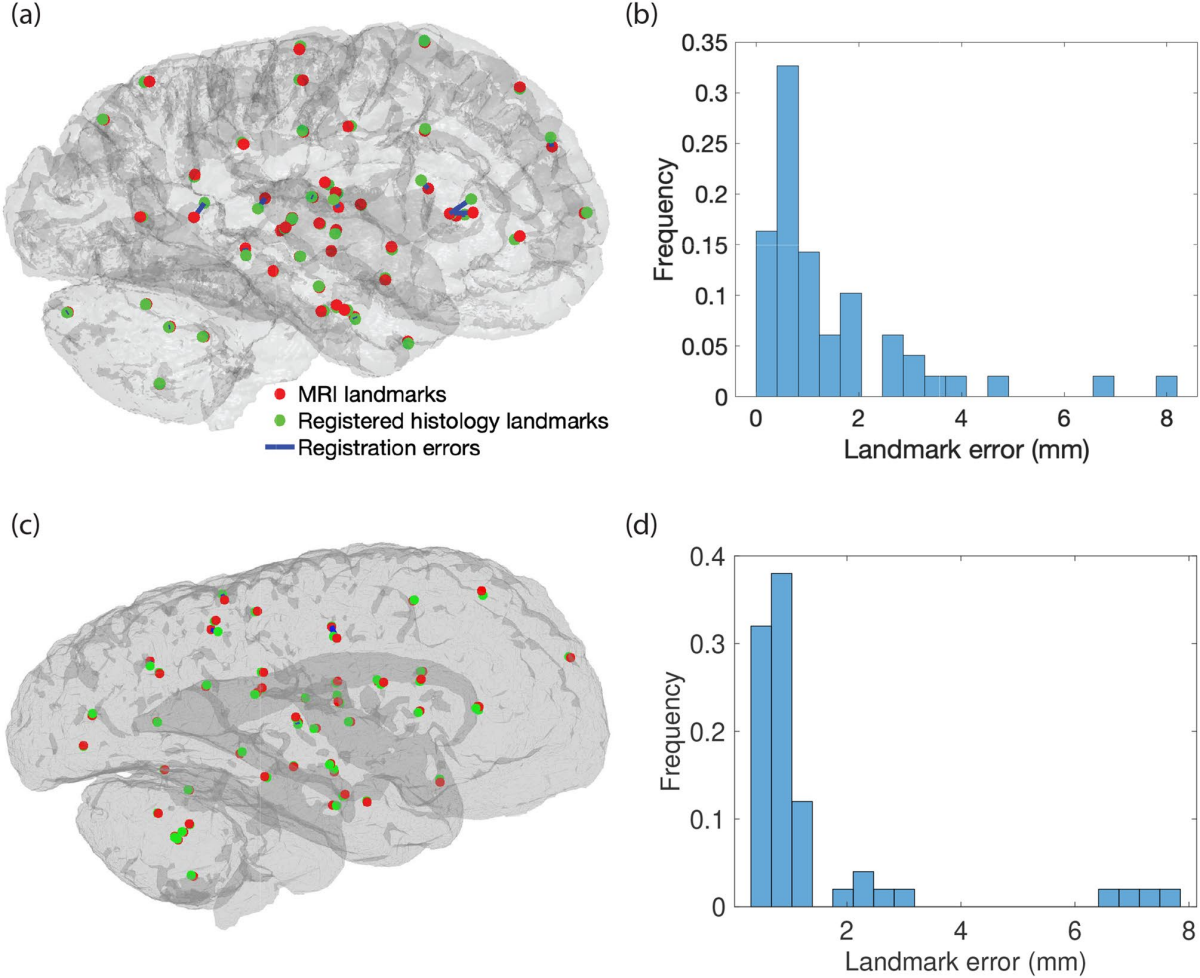
Registration error in 3D MRI-histology alignment. (**a**) 3D visualization of the landmark pairs after registration. The red spheres represent the landmarks annotated on the MRI. The green spheres represent the corresponding landmarks on the histology, after shifting them with the deformation field estimated by our registration algorithms. The blue lines connect landmark pairs, thus illustrating registration error. (**b**) Distribution of the landmark registration error (mean: 1.50 mm, standard deviation: 1.61 mm; median: 0.89 mm). (**c**,**d**) 3D visualization of landmark pairs after registration and distribution of the landmark registration error for the second processed hemisphere (mean: 1.44 mm; standard deviation: 1.80 mm; median: 0.80 mm).

Figure 8 shows the results for the landmark-based registration error, including a 3D visualization and a histogram with the distribution of the registration error. Both show that most landmarks are accurately registered in both hemispheres, with median errors equal to 0.8 and 0.89 mm, which is approximately two MRI voxels. When errors from the linear and nonlinear registration accumulate in similar directions, the error grows to a maximum of 8 mm, but 94% of the landmarks remain under 5 mm, which is approximately equal to the inter-rater variability reported in^77^.

**Figure 9:**
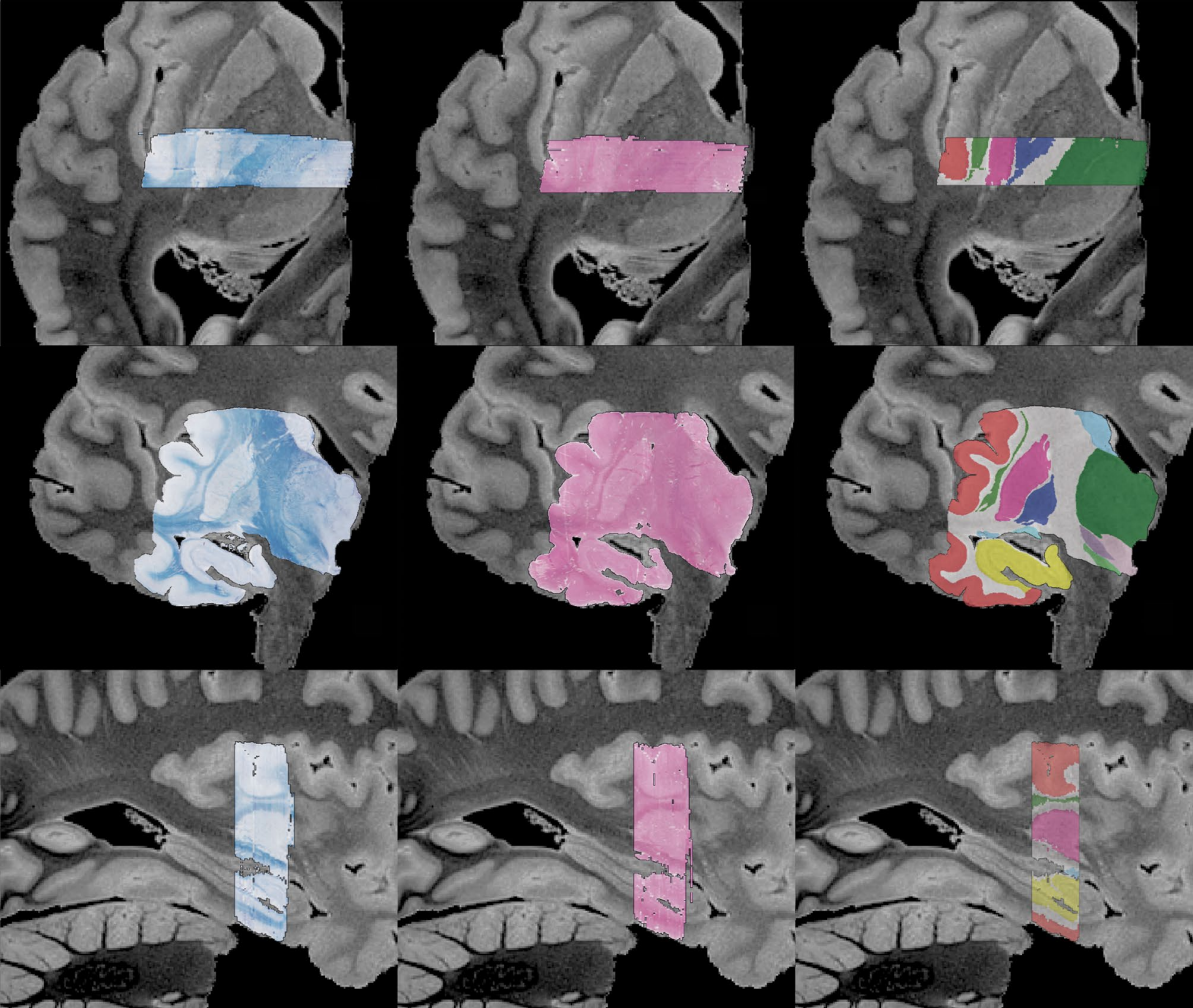
MRI-histology alignment. Histological sections from a sample block aligned with the MRI volume as a result of the refinement algorithm, showed from three different views (top: axial; middle: coronal; bottom: sagittal) and for the two stains (left: LFB; center: H&E). On the right, we have overlaid semi-automated segmentations of brain structures made on the LFB sections, including the hippocampus (yellow), cerebral cortex (red), cerebral white matter (white), pallidum (dark blue), putamen (bright pink), claustrum (green), thalamus (dark green), caudate (light blue), hypothalamus (light pink), substantia nigra (bright green), and subthalamic nucleus (violet).

**Figure 10:**
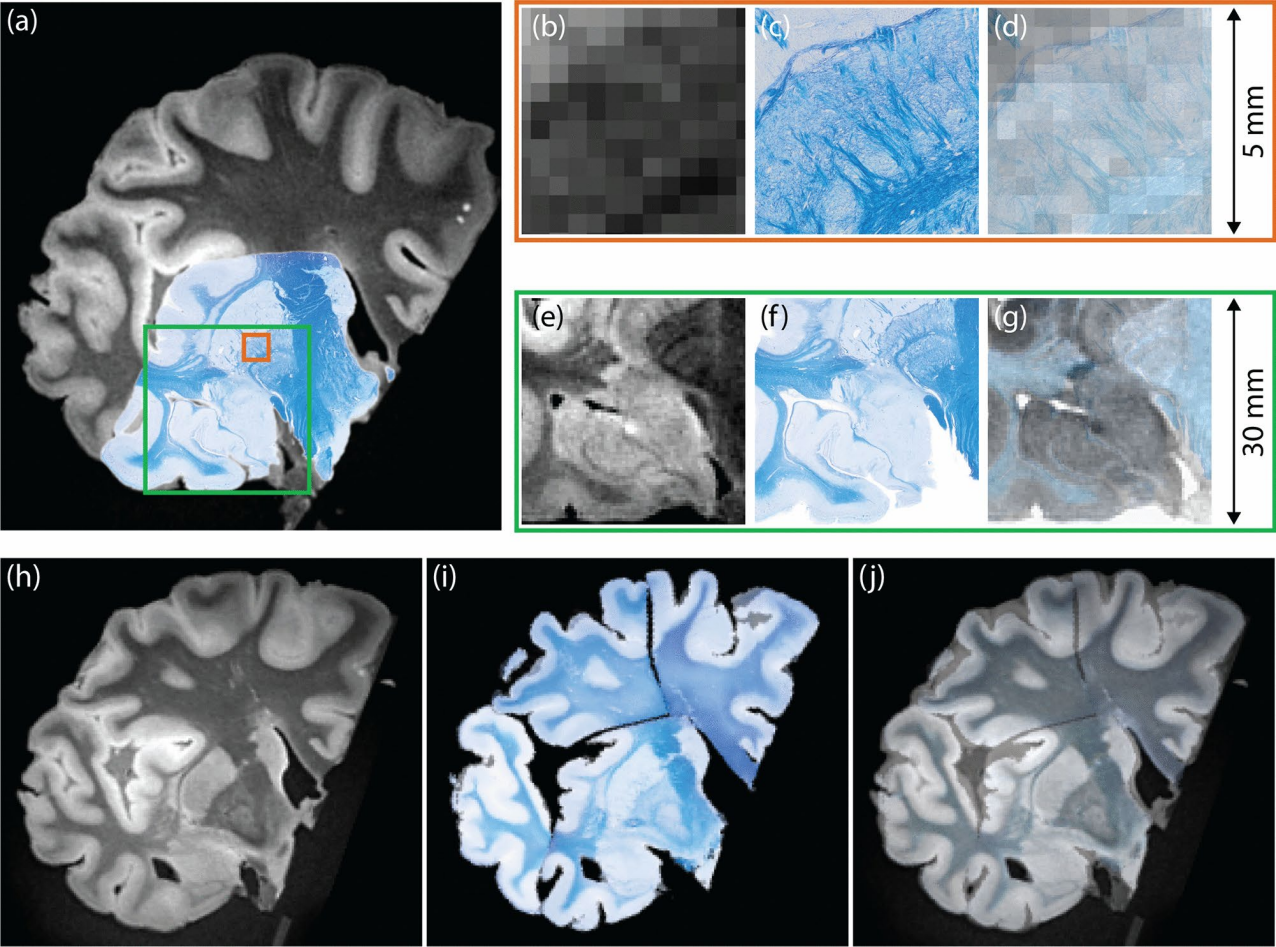
Registration between MRI and histological sections. (**a**) Sample MRI slice with registered LFB section overlaid; (**b**) magnified view of the 5 $\times$ 5 mm area marked in red; (**c**) corresponding region on LFB section; (**d**) MRI and LFB overlapped in transparency; (**e**–**g**) magnification of the 30 $\times$ 30 mm area marked with the green square in (**a**); (**h**) another sample coronal MRI slice; (**i**) corresponding slice in reconstructed LFB mosaic; (**j**) overlay of (**h**) and (**i**) in transparency.

### Navigating histological sections in 3D

Navigating through different scales of co-registered histology-MRI data, e.g., to localize and examine a section of interest, is one of the most immediate applications of this pipeline. Given the estimated transformations between blockface photographs and histological sections, it is possible to use the refined, MRI-aligned blockface mosaic to jointly explore the histological sections and the MRI in a common three-dimensional space. Figure 9 shows the same block as Fig. 6, this time overlaid on the MRI volume; for visualization, we also labeled whole brain structures on the LFB sections of this block with a semi-automated approach^78^, and deformed them along with the histology. The figure displays the alignment of the different brain structures across MRI and histology, both in the sectioning (coronal) and reconstructed planes (axial, sagittal), highlighting the smoothness of the reconstructed 3D histology and segmentations. Also in this case, similar results were obtained for the second hemisphere (Supplementary figure 2).

Figure 10a shows a more detailed example of navigation and alignment, displaying an LFB section on the corresponding resampled MRI plane. The red square delineates a 5 $\times$ 5 mm patch magnifying part of the lentiform nucleus (Fig. 10b–d), specifically the boundaries between the putamen and the two segments of the globus pallidus. The alignment is excellent, and the histology reveals details that are effectively invisible in MRI (even at sub-millimeter resolution). Likewise, the green square marks a 30 $\times$ 30 mm patch magnifying hippocampal head and the amygdala (Fig. 10e–g). Once again, the alignment between the boundaries across the two modalities is qualitatively very high.

Navigation is further exemplified in Supplementary video 2, which combines the multimodal imaging data with the segmentations from SAMSEG and FreeSurfer. Starting from the brain surface, the perspective slowly focuses on the basal ganglia, highlighting the alignment between blockface photograph, MRI and histology across different scales—from centimeters to microns per pixel. Finally, it is also possible to reconstruct an entire histology volume by mosaicking all the sections in 3D. An example at 0.4 mm resolution (i.e., the voxel size of the MRI) is showed in Fig. 10h–j.

## Discussion

In this paper, we have described a scalable and reproducible method for MRI-informed 3D histology. This includes both a protocol for tissue cutting and processing, without any requirements apart from a standard microtome, and a computational pipeline that requires minimal manual intervention. We have also presented results for single hemispheres from two human brains processed with the pipeline. As the reader may have observed, the proposed procedure consists of several steps, each with different design choices. In this section, we want to provide further context for such choices and discuss how they influenced our results.

### MRI as a reference for histology

The transition towards 3D histology is becoming necessary for a more complete study of biological specimens at a microscopic level. Such specimens are 3D objects and therefore a 3D characterization is required. Unfortunately, histology requires by definition the loss of 3D shape for the target object, and without prior information on the original shape, it is not possible to reconstruct a 3D volume in a way that is coherent with the original object.

As outlined in the introduction, MRI is a powerful tool when imaging entire organs, especially for large human organs such as the brain. There are virtually no comparable alternatives when one considers also how different acquisition sequences can be used to obtain a diverse collection of contrasts. Unfortunately, the wide spatial coverage comes at the expense of a coarser resolution. Although the idea of combining MRI and histology to achieve the best of both worlds is reasonable, the practical implementation is not straightforward. For instance, registration of a single histological section leads to a difficult slice-to-volume problem^8^, with the disadvantages of high sensitivity to initialization conditions and challenging multi-modal registration issues^6^. One issue to mention is the complicated assumption of the sectioning plane being parallel to MRI acquisition plane, that can be tackled using novel approaches based on 3D printed containers^48,50^ as mentioned in the introduction. This is why our approach relies on a volume-based approach, by first assembling together histological sections from the same block into volumes. In this way, although the mentioned 3D printed containers could simplify the computational processing, they are not an additional requirement. The overall registration problem still requires further steps as discussed in the following paragraphs.

### Choice of intermediate modality

An important choice in designing a pipeline for 3D histology regards the intermediate modality, which will serve as a stepping stone between the stained sections and the volumetric reference. In this paper, we relied on blockface photographs. Since they keep structural information before the cutting procedure^6^, they allow on one hand to assume a linear relationship with the reference, and on the other hand to deterministically map histological sections and blockface photographs. However, there are other potential candidates for this role, most notably MRI, OCT, PLI and clearing techniques.

MRI itself is a candidate for intermediate modality: one can scan individual blocks after the brain cut, and use them as stepping stone (e.g., as in^33^). While this strategy simplifies the registration of the blocks with the volumetric reference (since it becomes an intra-modality registration problem), it also has two disadvantages. First, it is considerably more expensive, since requires scanning the tissue twice. And second, the direct slice mapping between the intermediate modality and histology is lost, so one needs to estimate the relative orientation between the MRI of the block and the stack of histological sections; this is not necessary with blockface photography because every histological section has a known matching photograph.

OCT is an interferometry technique based on near-infrared light^14^. OCT volumes can be acquired during the sectioning procedure of a sample, with the important difference (compared with histology) that the data are acquired *before* sectioning. Therefore, geometric distortion is avoided and direct stacking of the 2D images yields a 3D consistent volume. While OCT produces images with excellent contrast at high resolution, it is much more costly to acquire than photographs, especially in terms of time^79,80^: imaging a single 20 mm thick human tissue block at $5 \times 5 \times 50\,\upmu$m takes several days of uninterrupted data acquisition.

Also based on optical imaging, PLI exploits the transmission of polarized light to give a quantitative estimate of fiber orientation and inclination angles for a given point in a tissue section^15^. This technique has great potential to study white matter at the mesoscopic and microscopic scales^81^, but still requires sectioning.

Tissue clearing^16^ is a powerful solution that avoids sectioning. Clearing methods can make opaque tissue transparent and, combined with fluorescent labeling tools, they offer a new way of probing microscopic structures^82,83^. As a consequence of the transparency, there is no need to slice the sample and it is sufficient to adjust the focus of the microscope on the plane of interest. The main drawbacks of this technique are the long time needed to process the tissue, the size limits for clearable samples and the limited depth of antibody penetration in the cleared tissue.

It is evident then that the choice of an intermediate modality is a trade-off between result quality and costs. Therefore, we chose to use blockface photographic imaging as it is cheap and fast, and as a result ideal for larger scale studies. The poorer contrast of blockface photographs compared to the alternatives is indeed a limit of the approach presented here, but in the context of the whole pipeline, it serves its purpose well as intermediate modality.

### Assumptions of registration-based pipelines

In our pipeline, we have made the assumption that the deformation between the MRI and the tissue blocks (imaged via photography) is linear. This is an approximation: even with fixed tissue, cutting into slices and blocks introduces small nonlinear deformations, particularly near the cut boundaries. Moreover, tissue processing also introduces nonlinear distortions, even if minimal.

Another potential error source for the registration is the imperfection of the automated segmentation of the blockface photographs, particularly when the tissue is concave: since paraffin is not opaque, the apparent surface of the tissue on the blockface is often overestimated. As these automatically generated masks are crucial in the regularization of our linear registration, their oversegmentation may have directly affected the quality of our linear alignment and thus our results. This problem could be mitigated by integrating the linear and nonlinear registration algorithms: given the nonlinear registration of the histological sections, we could take advantage of the superior contrast of the stained sections and their more accurate automated segmentations, in order to improve the linear registration. In a similar fashion, the newly improved linear alignment could be used to refine the nonlinear registration, and so on. This approach may be particularly useful in order to avoid the overlap of tissue from different blocks in the reconstruction, and also to better fit the boundary of the tissue on the brain surface. Future work will explore this direction.

### Improving nonlinear registration

The final step of this pipeline and the last transformation needed for MRI-histology alignment is given by the registration between the resampled MRI slices and the related histological sections. In this case, the nonlinearities are considerable, and the cross-modality nature of the problem requires the use of inter-modality metrics, such as mutual information. As it has been previously shown^84^, approaches based on mutual information often perform poorly in inter-modality registration, and therefore represent a bottleneck when registering MRI images and stained sections—even when they are already initialized with our joint linear registration method. In order to improve the alignment, an important direction to explore is the use of synthesis, i.e. estimating MRI contrast from the histology (or vice versa) to reduce the registration to an easier intra-modality problem. Recent advances with architectures based on generative adversarial networks have shown great potential for this specific problem^85,86^, even with specific applications to medical imaging^87^.

### Future applications

In this paper we have presented, as a preliminary practical application of our pipeline, the chance of exploring histological sections through the related MRI volume. This is only the tip of the ice for the potential applications of our approach. In the overarching strategy of our current project, the next step is to acquire a larger set of brains, manually segment the stained sections, and exploit 3D histology to create a probabilistic atlas of the human brain at the subregion level. The MRI not only serves as frame of reference for the reconstruction, but can also be used to parcellate the cortex. This parcellation can be used to include cortical regions in the probabilistic atlas, and also to aid the segmentation of cortical structures in histological sections.

As opposed to existing techniques, our pipeline will be able to build whole-brain datasets from stained sections, allowing us to build atlases that are much more detailed than current templates. Moreover, since specific staining agents and immunohistochemistry techniques can enhance different microscopic details, the approach described here opens the door to draw new multimodal maps of the human brain^88^, in a scalable and reproducible way. With the advancement of microscopy-oriented techniques (e.g.,^50,82,89–92^), we believe that closing the gap with macroscopic modalities is crucial.

Another potential application is the use of histology for development of MRI-based biomarkers and quantitative imaging^93^: as ex vivo validation with histology becomes more common, the neuroimaging field will largely benefit from 3D histology.

Finally, aligned MRI and histology also have the potential to lead to ultra-large-scale microscopy: so far it is possible to create a large dataset with several images acquired using electron microscopy, by stitching them together to cover the entire histological section^94^. In this perspective, our approach could potentially lead to the ability to navigate a brain volume and then retrieve ultra-high-resolution details from a point of interest. This would be the new frontier of multi-scale imaging and would of course create new challenges, since the associated storage and processing requirements are highly demanding.

### Conclusion

We have presented a pipeline to effectively obtain high-resolution 3D images of the human brain using histology and MRI. The related code and the acquired data are publicly available (http://www.jeiglesias.com), and we plan to use the presented methods to build a high-resolution computational atlas of the human brain based on 3D reconstructed histology. As increasingly more advanced macroscale and microscale techniques to study the brain become available, the open-source tools we have presented here will have a key role in bridging together the two ends of the scale.

## Electronic supplementary material

Supplementary Information 1.

Supplementary Video 1

Supplementary Video 2
