# Supplementary material for: A multimodal computational pipeline for 3D histology of the human brain
Source: Sci Rep. 2020 Aug 14;10:13839. doi: 10.1038/s41598-020-69163-z (PMC7429828; doi:10.1038/s41598-020-69163-z)
Supplement: Supplementary file 1 — Supplementary Information 1. [file 41598_2020_69163_MOESM1_ESM.pdf]

A multimodal computational pipeline for 3D histology of the human brain - Supplementary materials

Matteo Mancini[1,2,3,4,*], Adrià Casamitjana[1], Loic Peter[1], Eleanor Robinson[1,5], Shauna Crampsie[5], David L. Thomas[6,7], Janice L. Holton[5], Zane Jaunmuktane[5], Juan Eugenio Iglesias[1,8,9,*]

1 - Centre for Medical Image Computing, Dept. Medical Physics & Biomedical Engineering, University College London, London, United Kingdom
2 - Department of Neuroscience, Brighton and Sussex Medical School, University of Sussex, Brighton, United Kingdom
3 - CUBRIC, Cardiff University, Cardiff, United Kingdom
4 - NeuroPoly Lab, Polytechnique Montreal, Montreal, Canada
5 - Queen Square Brain Bank for Neurological Disorders, UCL Queen Square Institute of Neurology, University College London, London, United Kingdom
6 - Neuroradiological Academic Unit, UCL Queen Square Institute of Neurology, University College London, London, United Kingdom
7 - Leonard Wolfson Experimental Neurology Centre, UCL Queen Square Institute of Neurology, University College London, London, United Kingdom
8 - Athinoula A. Martinos Center for Biomedical Imaging, Massachusetts General Hospital and Harvard Medical School, Boston (MA), USA
9 - Computer Science and Artificial Intelligence Laboratory (CSAIL), Massachusetts Institute of Technology, Cambridge (MA), USA

*** For correspondence:**
m.mancini2@bsms.ac.uk (MM)
e.iglesias@ucl.ac.uk (JEI)

Supplementary figures

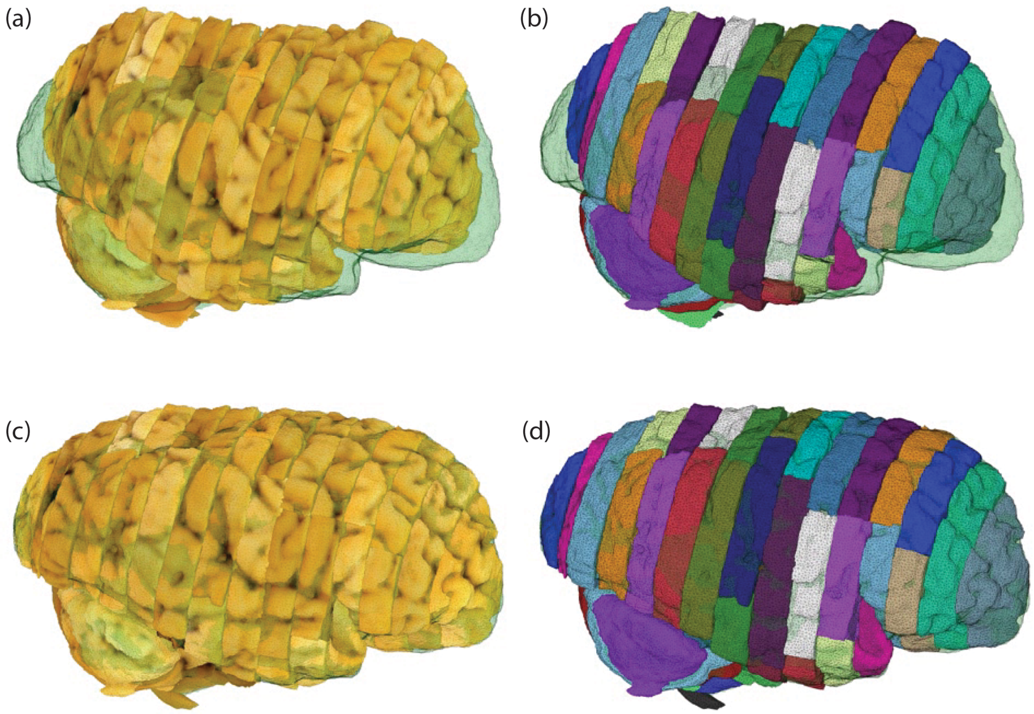

Supplementary figure 1: **Initialization and refined blockface mosaics for the second processed hemisphere.** A surface rendering of the whole brain mask derived from the MRI is overlaid in green. (a) Blockface volume for the second hemisphere, resulting from the initialization with the slice photographs; the color for rendering is taken from the blockface photographs. (b) Same volume as (a), where each block is rendered in a different color. The color coding is random and simply emphasizes the block cutting profiles. (c,d) Same volumes as (a,b) after joint registration.

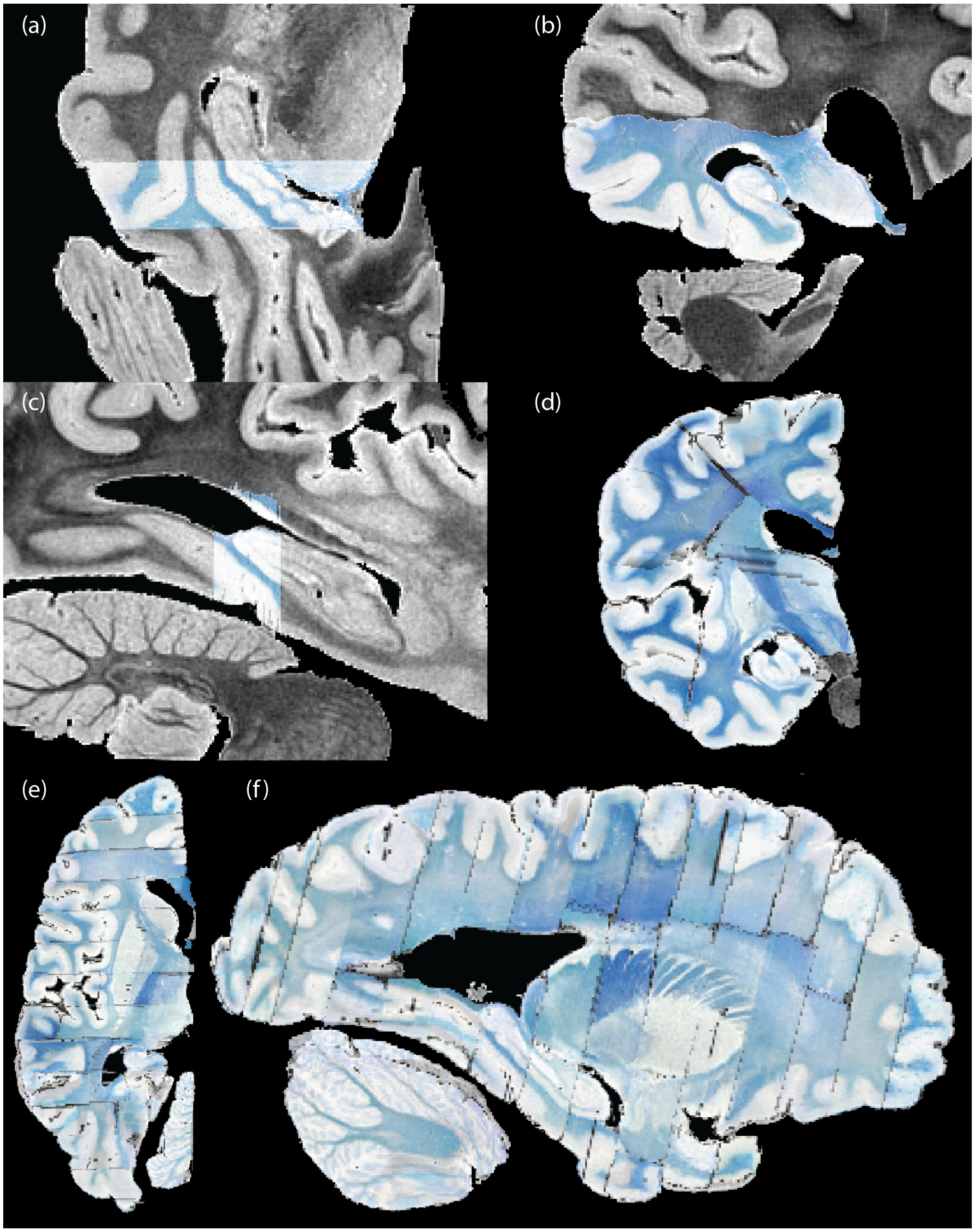

Supplementary figure 2: **MRI-histology alignment for the second processed hemisphere.** (a-c) Histological sections (LFB stain) from a sample block aligned with the MRI volume as a result of the refinement algorithm, shown for three different views (a - axial; b - coronal; c - sagittal). (d-e) Sample MRI slices with registered LFB sections overlaid (d - coronal; e - axial; f - sagittal).
